# Simultaneous Measurement of Microdisplacement and Temperature Based on Balloon-Shaped Structure

**DOI:** 10.3390/s23208521

**Published:** 2023-10-17

**Authors:** Yaxun Zhang, Yuxin Liu, Zhiliang Huang, Pingbang Huang, Xiaoyun Tang, Zhihai Liu, Yu Zhang, Libo Yuan

**Affiliations:** 1 Qingdao Innovation and Development Center, Harbin Engineering University, Qingdao 266500, China; 2 Key Lab of In-Fiber Integrated Optics, Ministry Education of China, Harbin Engineering University, Harbin 150001, China; 3 Key Laboratory of Photonic Materials and Device Physics for Oceanic Applications, Ministry of Industry and Information Technology of China, Harbin Engineering University, Harbin 150001, China; 4 College of Shipbuilding Engineering, Harbin Engineering University, Harbin 150001, China; 5 College of Photonic and Electronic Engineering, Guilin University of Electronic Technology, Guilin 541004, China

**Keywords:** core-offset joints, balloon-shaped single mode fiber, multimode interference, microdisplacement, temperature sensor

## Abstract

An optical fiber sensor for the simultaneous measurement of microdisplacement and temperature based on balloon-shaped single-mode fibers cascaded with a fiber Bragg grating with two core-offset joints is proposed. The interference between the core mode and cladding mode is caused by the stimulation of the cladding mode by the core-offset joints’ structure. The cladding of the core has a distinct refractive index, which causes optical path differences and interference. The balloon-shaped structure realizes mode selection by bending. As the displacement increases, the radius of the balloon-shaped interferometer changes, resulting in a change in the interference fringes of the interferometer, while the Bragg wavelength of the fiber grating remains unchanged. Temperature changes will cause the interference fringes of the interferometer and the Bragg wavelength of the fiber grating to shift. The proposed optical fiber sensor allows for the simultaneous measurement of microdisplacement and temperature. The results of the experiment indicate that the sensitivity of the interferometer to microdisplacement is 0.306 nm/µm in the sensing range of 0 to 200 μm and that the temperature sensitivity is 0.165 nm/°C, respectively. The proposed curvature sensor has the advantages of a compact structure, extensive spectrum of dynamic measurement, high sensitivity, and simple preparation, and has a wide range of potential applications in the fields of structural safety monitoring, aviation industry, and resource exploration.

## 1. Introduction

Microdisplacement sensors have a high level of measurement precision, such as conventional interferometers, eddy current sensors, and optical fiber sensors, amongst others [1,2]. However, interferometers [3] are difficult to implement due to their size and complexity. Eddy current sensors [4] are susceptible to electromagnetic interference and possess a low degree of precision. Compared with the conventional microdisplacement sensor, the optical fiber sensor has the advantages of a high integration and good anti-interference performance. Fiber-based microdisplacement sensors typically use grating structures, including fiber Bragg gratings (FBG) [5,6,7], long-period fiber gratings (LPFG) [8], and tilted gratings [9]. In addition, there are optical-fiber microdisplacement sensors based on different interference principles, including multimode interference (MMI) [10], Mach–Zehnder interference (MZI) [11], Fabry–Perot interference (FPI) [12,13,14], etc. In comparison to grating microdisplacement sensors, optical fiber sensors based on the interference principle offer a greater measurement precision and wider measurement range.

In order to increase the sensitivity of optical fiber sensors, various configurations of optical fiber sensors with offset core structures have been proposed [15,16]. The structure is spliced by the offset axis of the fiber core and the cladding, causing interference between the cladding mode and the core mode. By utilizing a single-mode fiber with an offset core, Wen et al. [17] measured the refractive index with a high sensitivity. However, the mechanical properties of the sensor are insufficient due to the large offset of the offset core. Qiu et al. [18] manufactured refractive index (RI) sensors by clamping the hollow fiber (HCF) section between the tail fiber introduced into the single-mode fiber (SMF) and the reflected SMF section to enhance the device’s mechanical properties. Similar structures were used by Huang et al. [19] to simultaneously measure strain and temperature. The structure of the sensor is compact, and the length of the device can be further reduced. Zhang [20] improved the Mach–Zehnder interference, accomplished torsion sensing, and decreased the cross sensitivity between torsion and temperature by employing the offset core construction. Gu et al. [21] addressed the core shift sensor for biomolecule detection based on SMF and multimode fiber. The findings demonstrate the significant potential of the core offset structure in the creation of optical fiber sensors for biological detection. The optical fiber’s bending structure can also increase the sensitivity of the optical fiber sensor [22]. This method is extensively used to measure physical parameters, such as the refractive index [23,24,25], magnetic field [26], curvature [27], temperature [28], and salinity [29]. Combining the dislocation and bending structure of an optical fiber was previously difficult to achieve in the previous way. To achieve mode selection, we bring bending into dislocation. The decoupling method of the dual-parameter measurement of the optical fiber sensor can be decoupled by the machine-learning method in addition to the sensitivity transfer matrix [30]. The difference between the two is that the transfer matrix method theoretically obtains an accurate solution, while machine learning requires a lot of learning [31,32,33].

In this article, we present a fiber sensor for the simultaneous measurement of microdisplacement and temperature, which is composed of core-offset-joints (COJs)-based balloon-shaped SMF and FBG with two. The COJs are realized by splicing three single-mode fibers with a minor lateral offset. Due to this design offset, the interference will occur between the core mode and the cladding mode. A balloon-shaped structure is used to select the interference mode and form the Mach–Zehnder interference. The FBG cascaded with spherical SMF with COJs can sense temperature changes. The spectral pattern of the device will change when the displacement and/or temperature of the ambient material vary. The theoretical analysis is consistent with the experimental results. The results indicated that this novel sensor has the advantage of a simple configuration, low cost, easy operation, high sensitivity and stability.

## 2. Theory and Sensing Principle

**Figure 1:**
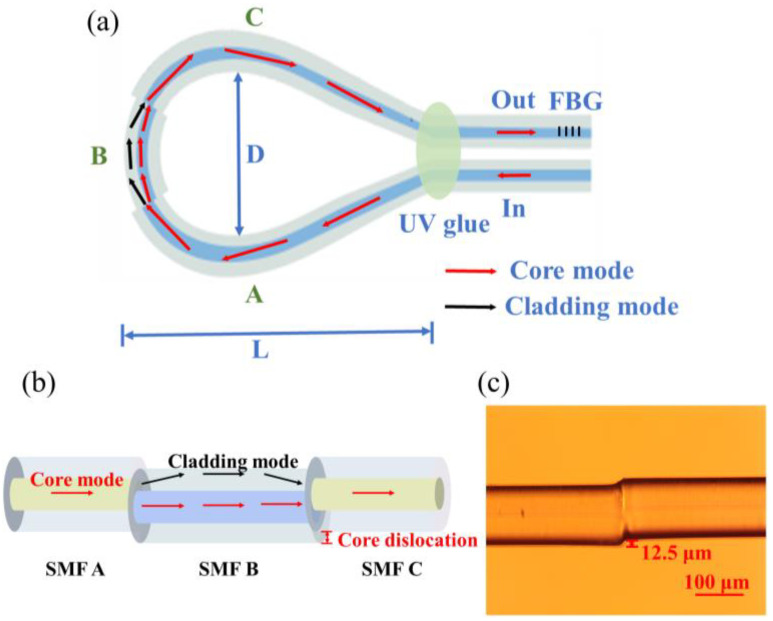
(**a**) Schematic diagram of the COJ-based balloon-shaped single-mode fiber structure, formed by three single-mode fibers (A, B, and C); (**b**) cross offset structure; (**c**) microscope image of a single-mode fiber COJ along the fiber axial direction.

Figure 1a shows a schematic diagram of the balloon-shaped core-offset bending SMF fiber structure. The values D and L represent the bending diameter and breadth of the structure, respectively. The SMF structure with a core offset is centered on top of the balloon structure.

As seen in Figure 1a, we realize partial cladding mode excitation by cascading bending. Due to the COJ structure in Figure 1b, the light is separated into two pathways: one enters the core of the SMF B, and the other is sent into the cladding as a cladding mode. After propagating for a distance in the SMF B, the light in the cladding enters the multimode SMF C and can be coupled back to the core. Two fiber core-offset structures generate MZI interference owing to the phase mismatch between the cladding mode and the core mode. The transfer function of MZI is expressed as:

(1) $$I=I_{1}+I_{2}+2\sqrt{I_{1}I_{2}}\cos\left(2\pi \Delta nd/\lambda \right)$$

where *I* is the intensity of the interference signal. *I_1_* and *I_2_* are the intensities of the core and cladding modes. Δ*n* is the effective refractive index difference between the core mode *n_1_* and the cladding mode *n_2_*. *d* is the interferometer arm length, whereas *λ* is the operational wavelength. Therefore, the reduction in transmission spectrum wavelength *λ_k_* may be stated as:

(2) $$\lambda_{k}=2\Delta nd/\left(2k+1\right)$$

The interval between consecutive peaks is approximately *λ^2^/*Δ*nd*. Multimode interference makes up the majority of fiber MZI. In this structure, the cladding of the SMF can be regarded as a multimode waveguide. Due to the core-offset splicing approach, a beam from a portion of the core spreads to the cladding, generating an MZI with more than one mode. Since each mode responds differently to changes in the environment, this multimode interference affects the sensing performance. In order to control the generated cladding mode, we achieve mode selection by bending part of the core-offset connection. The bent offset core structure will cause *n* to alter, which will cause *k* to vary as well. We use spectral drift to calculate the microdisplacement. The RI distribution in the COJ-based balloon-shaped SMF structure can be expressed as [22]:

(3) $$n^{\prime }=n_{0}\left(1+\frac{x}{r_{eff}}\right)$$

where *x* is the normal axis of the bending fiber, *n_0_* is the RI of the straight fiber section, and *r_eff_* is the equivalent bending radius, which may be computed by [24]:

(4) $$r_{eff}=\frac{r}{1-\frac{n_{0}^{2}}{2}\left[P_{12}-\upsilon \left(P_{11}+P_{12}\right)\right]}$$

where *r* is the actual bending radius of the fiber, and *ν* is the ratio of Poisson. *P*_11_ and *P*_12_ are the photoelastic tension of each component. The bending causes the refractive index to shift. In general, RI decreases to the outside of the bending.

The characteristic of FBG is that the RI of the optical fiber core can be periodically modulated. The temperature response of FBG is mainly affected by the thermal dependence of RI [33]. Therefore, FBG can resonate with the Bragg wavelength *λ_FBG_*, which is determined by the effective RI of the fiber core *n_eff_* and the grating period *Λ*. The *λ_FBG_* can be expressed as [7]:

(5) $$\lambda_{FBG}=2n_{eff}\Lambda$$

Based on the foregoing theoretical study, (2) demonstrates that, in principle, the longer the SMF B, the better. However, in the experiment, the mechanical strength of the sensor will be diminished due to the excessive length of the SMF B. After calculation and experimental verification, the 25-mm-length sensor can demonstrate a satisfactory performance. The core/cladding diameter and RI of SMF are 8.2/125 μm and 1.4446/1.4447, respectively. The amount of core dislocation *z* is 12.5 μm.

## 3. Experimental Results

### 3.1. Experimental Setup

To fabricate the balloon-shaped core-offset bending SMF fiber structure shown in Figure 1, two core-offset structures were fabricated on SMF (Corning SMF-28, New York, NY, USA) using a commercial fiber fusion splicer (Fujikura-88S, Tokyo, Japan). The direction between the two COJ-based structures are irrelevant, which simplifies the production process. The two COJ-based structures have an offset of around 12.5 μm. Figure 1c depicts the microscopic view of a single offset core structure. The bending radius R of the fabricated sample is 12.83 mm to obtain the best interference deflection extinction ratio, and the corresponding width of the balloon-shaped section L is 65.92 mm. FBG is connected in series with the fiber structure.

**Figure 2:**
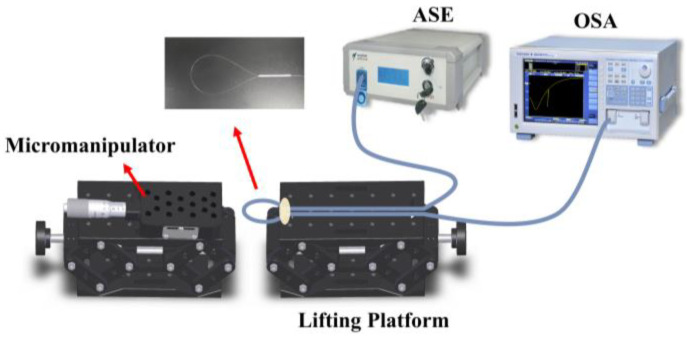
Schematic diagram of the experimental setup for displacement measurement using the proposed sensor.

The displacement measurement experimental setup is depicted in Figure 2. The height of the lifting platform is adjustable, and ultraviolet (UV) glue is used to adhere the sample to the platform. The micromanipulator (MPC-385, Sutter, Atlanta, GA, USA) is utilized to manually modify the translation stage’s position. The accuracy of the displacement adjustment is 1 µm, which adjusted the axial displacement and applied it to the balloon-shaped section precisely. In order to describe the device, a broadband light source (ASE, Hoyatek, Shenzhen, China) is emitted into it, and an optical spectrum analyzer (OSA, AQ6370D, Yokogawa, Tokyo, Japan) with a maximum spectral resolution of 0.02 nm is utilized to monitor the transmission spectrum.

### 3.2. Results and Discussion

**Figure 3:**
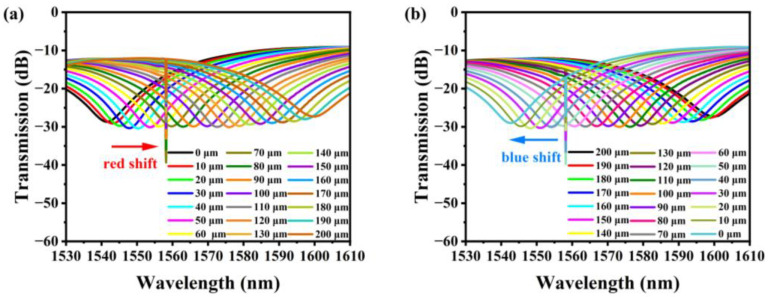
The development of dip A as displacement varies: (**a**) displacement increases (0–200 μm); (**b**) displacement decreases (0–200 μm).

As depicted in Figure 3a, there is a desired interference dip angle between 1530 and 1610 nm. There are two reasonably significant extinction ratio dips in the recorded spectrum for the later measurement: the resonance wavelength induced by FBG at 1558.16 nm (dip B) and the resonance wavelength of MZI at 1540.14 nm (dip A).

The transmission spectrum of the fiber structure varies when the displacement is between 0–200 μm and the step size is 10 μm. In order to evaluate the repeatability of the sample sensor, the displacement is decreased from 200 μm to 0 μm and the measurement process is performed in reverse. The results are displayed in Figure 3b. As depicted in Figure 3a, when the displacement increases, the inclination angle at 1540.14 nm is red-shifted, while the inclination extinction ratio remains nearly unchanged. As depicted in Figure 3b, the spectral evolution is reversed when the displacement direction changes. Due to the peculiarity of the Bragg grating resonance wavelength, when the displacement varies, the wavelength of dip B remains unchanged at 1558.14 nm. The variation in the dip A wavelength is due to the form change of the balloon-shaped cross section produced by displacement, which ultimately results in the variation of the effective bending length *r_eff_* and the variation of optical coupling between the SMF core and cladding. During the experiment, the inclination extinction ratio will fluctuate slightly due to shifts in temperature, humidity, etc.

**Figure 4:**
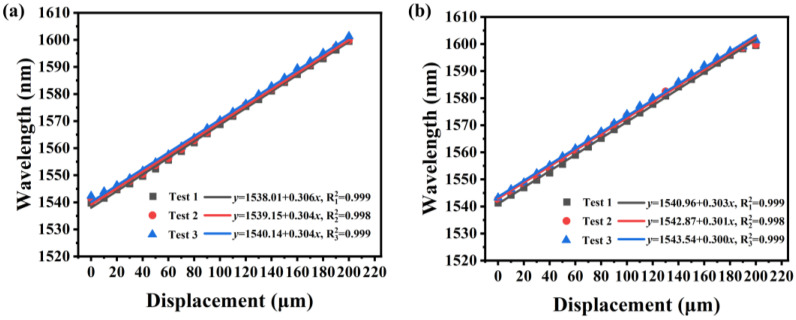
The results of the sample measurement repeatability test show that the spectral wavelength shift is a function of displacement, including (**a**) displacement increase and (**b**) displacement decrease.

The wavelength shift and intensity change of dip A with increasing and decreasing displacement are linearly fitted in Figure 4a,b, respectively. The wavelength-displacement sensitivity of dip A to the displacement-increasing process may be computed from Figure 4a as reaching 0.306 nm/μm, and the linear regression coefficient (R^2^) achieves 0.999. The sensitivity of dip A to the displacement-reduction procedure can approach 0.303 nm/μm, and R^2^ is 0.999, as can be seen in Figure 4b. This sample also demonstrates that there is almost no hysteresis in the sensor and that the displacement measurement has good repeatability: the outcomes of three separate experiments are virtually identical.

**Figure 5:**
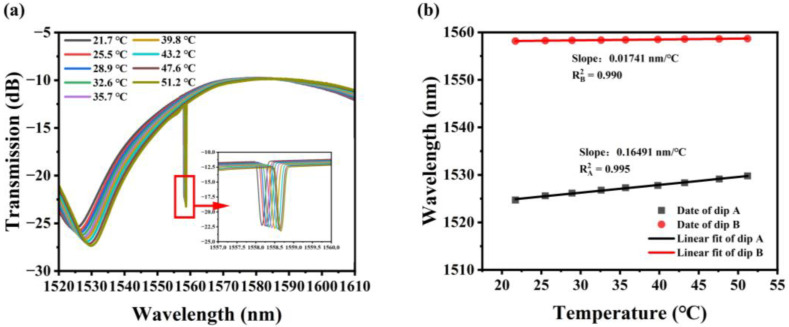
When the temperature changes, the sensor (**a**) changes in terms of the transmission spectrum; (**b**) linear fitting of the center wavelength drift of dip A and dip B.

In addition to measuring displacement, the temperature dependence of the pro-posed fiber structure was investigated by placing the sensor sample on a heating plat-form. The temperature measurement range is 21.7–51.2 °C due to the limited operating temperature range of the UV glue. The transmission spectrum of the sensor changes when the temperature changes, as shown in Figure 5a. Redshift happens as dips A and B both drift to longer wavelengths as the temperature rises. Figure 5b depicts the displayed and fitted measured data. According to the findings of the linear fitting, dip A generated by MZI has a temperature sensitivity of 0.165 nm/°C, and dip B induced by FBG has a temperature sensitivity of 0.017 nm/°C, with R^2^ values of 0.995 and 0.990, respectively. It is worth noting that dip A in this structure has a temperature sensitivity that is 9.47 times greater than dip B, indicating that the MZI interference caused by the curved offset core structure is temperature-sensitive.

The wavelength shift of the dip A transmission spectrum is composed of temperature change and displacement change. Based on the experimental findings described above, the displacement and temperature changes of the sensor can be described by the following matrix:

(6) $$\left[\begin{array}{c} \Delta \lambda_{dipA}\\ \Delta \lambda_{dipB} \end{array}\right]=\left[\begin{array}{cc} K_{dipA}^{\Delta x} & K_{dipA}^{T}\\ 0 & K_{dipB}^{T} \end{array}\right]\left[\begin{array}{c} \Delta x\\ \Delta T \end{array}\right]$$

The wavelength changes of the offset-core bent MZI and FBG are represented by ∆*λ_dipA_* and ∆*λ_dipB_*, respectively. The displacement sensitivity and temperature sensitivity of MZI are denoted by $K_{dipA}^{\Delta x}$ and $K_{dipA}^{T}$. The temperature sensitivity of FBG is $K_{dipB}^{T}$. The changes in temperature and displacement are denoted by Δ*x* and Δ*T*. The variation of temperature and displacement can be expressed using the below matrix:

(7) $$\left[\begin{array}{c} \Delta x\\ \Delta T \end{array}\right]={\left[\begin{array}{cc} K_{dipA}^{\Delta x} & K_{dipA}^{T}\\ 0 & K_{dipB}^{T} \end{array}\right]}^{-1}\left[\begin{array}{c} \Delta \lambda_{dipA}\\ \Delta \lambda_{dipB} \end{array}\right]$$

Additionally, considering that the OSA utilized in the experiment has a resolution of 0.02 nm, the sensor system’s displacement measurement resolution is 1 μm, and the temperature measurement precision is 0.1 °C, all of which are acceptable for usage in actual applications.

The proposed sensor is compared to displacement and temperature sensors with various optical setups that have been previously described. The sensors using wavelength demodulation have a high sensitivity and large measuring range, as shown in Table 1. According to Table 1, our sensor has a higher sensitivity than those from earlier research. The sensitivity of our sensor is much higher than that of the same type of temperature and displacement dual-parameter measurement sensor with a balloon shape. Our work’s displacement sensitivity is 182.14 times higher than its displacement sensitivity and 5.77 times higher than its sensitivity to temperature [22]. In addition, the sensor benefits from an easy construction, high reproducibility, and excellent stability. The optical fiber sensor has great application potential in the field of optical fiber sensing, since it can simultaneously monitor displacement and temperature.

## 4. Conclusions

In this article, we propose and investigate a COJ-based balloon-shaped structure cascaded with an FBG-based simultaneous microdisplacement-and-temperature-measurement optical fiber sensor. The interference between the core mode and cladding mode is caused by the stimulation of the cladding mode by the core-offset joints’ structure. The balloon-shaped structure realizes mode selection by bending. Temperature changes will cause the interference fringes of the interferometer and the Bragg wavelength of the fiber grating to shift. The results indicate that the displacement sensitivity of the sensor is 0.306 nm/μm and that its measuring range is 0–200 μm. The temperature sensitivity in the range of 21.7–51.2 °C is 0.165 nm/°C. The suggested sensor further benefits from easy manufacturing, high reliability, low cost, and good repeatability.

## Figures and Tables

**Table 1:** Comparison of the performance of several sensors for temperature and displacement measurement.

Measurement Configuration	Temperature Sensitivity	Displacement Sensitivity	Refs.
Bent fiber with LPG	0.0429 nm/°C Range: 20–45 °C	−0.306 nm/μm Range: 0–80 μm	[34]
Bent fiber with FBG	0.105 nm/°C Range: 20–70 °C	0.18 nm/μm Range: 0–70 μm	[35]
Bent core-offset MZI	0.11745 nm/°C Range: 25–60 °C	-	[36]
FBG	-	0.567 pm/μm Range: 0.5–3.5 mm	[37]
MZI	0.0286 nm/°C Range: 15–35 °C	1.68 nm/mm Range: 0–5 mm	[22]
MMI	0.0116 nm/°C Range: 20–50 °C	0.00589 nm/μm Range: 0–600 μm	[38]
Bent core-offset MZI with FBG (This work)	0.165 nm/°C Range: 21.7–51.2 °C	0.306 nm/μm Range: 0–200 μm	-

## Data Availability

Not applicable.
